# A new design for the review and appraisal of semi-solid dosage forms: Semi-solid Control Diagram (SSCD)

**DOI:** 10.1371/journal.pone.0201643

**Published:** 2018-09-07

**Authors:** Anna Nardi-Ricart, Maria Jose Linares, Faviola Villca-Pozo, Pilar Pérez-Lozano, Josep Maria Suñé-Negre, Lara Bachs-deMiquel, Manel Roig-Carreras, Marc Suñé-Pou, Isaac Nofrerias-Roig, Encarna García-Montoya

**Affiliations:** 1 Pharmacy and Pharmaceutical Technology, and Physical Chemistry Department, Universitat de Barcelona, Barcelona, Spain; 2 IDIBELL-UB Research Group, Pharmacotherapy, Pharmacogenomics and Pharmaceutical Technology, L'Hospitalet de Llobregat, Barcelona, Spain; 3 Anesthesiology Service of Viladecans Hospital, Viladecans, Barcelona, Spain; 4 IDIBELL-UB Research Group, Patología del Tubo Digestivo, L'Hospitalet de Llobregat, Barcelona, Spain; Koc University, TURKEY

## Abstract

The Semi-solid Control Diagram (SSCD) is a new tool designed for the study of different excipients and different semi-solid dosage forms. It can be used to review and evaluate different formulations and/or batches and facilitate the selection of one of them that will present the most suitable galenic characteristics for topical application. It is also useful to track stability studies by comparing the diagrams, which allows to measure the impact of subjecting the formulation to different conditions and times to be examined. In this study, the Semi-solid Control Diagram (SSCD) is used as an instrument for studying and evaluating semi-solid pharmaceutical dosage forms, by comparing several different semisolid preparations (lipogels). With these results, the tool is validated and the best formulation has been discriminated from the others.

## Introduction

There are a large number of semisolid preparations, corresponding to different pharmaceutical dosage forms differentiating each other by their consistency and rheological characters. These forms are normally presented in the form of creams, gels, emulsions, ointments or pastes [1].

Water in oil (W/O) creams are lipophilic emulsions in which the external hydrophobic phase is a gel (hydrocarbon gel, oleogel or lipogel). This provides the formulation its good spreading properties [2].

They contain one or more active ingredients dissolved or uniformly dispersed in a suitable base and any suitable excipients such as emulsifiers, viscosity increasing agents, antimicrobials agents, antioxidants or stabilizing agents [3].

Some of the parameters that are usually studied to evaluate the quality of a semisolid formulation are Organoleptic properties, Viscosity. Extensibility, and Centrifugation [4][5][6]. These parameters are usually considered individually to establish the final quality of the product.

The new design, Semi-solid Control Diagram (SSCD), is a new proposed tool that has been designed for our lab team for reviewing and evaluating the quality of different semi-solid dosage forms, first determining the common parameters [4] that provide us information to define the quality of a semisolid formulation and secondly combining them to obtain an index of quality which helps the formulator to discriminate the best formulation.

It enables a standardized comparison between several references and/or batches to facilitate the selection of a formulation that will present the most suitable galenic characteristics for topical application.

This tool has been designed using the experience previously gained from applying the SeDeM system to evaluate the suitability for direct compression of active ingredients or excipients in powder form [7], to control batch powder formulation and preformulation of drug products [8], to develop direct compression tablet formulations [9][10][11][12], to define a classification of directly compressible excipients [13] and to develop Oral Dispersable tablets [14][15], modified-release tablets and determine the design space of the formula [16].

This tool could be broadly useful for formulation studies since it allows a comparison of the effect of the addition of excipients on the formulation or, for example, the comparative studies of the same excipient from different suppliers to be performed.

Another application for this tool is to evaluate and track stability studies of semi-solid formulations by comparing the diagrams, which allows measure the impact of subjecting the formulation to different conditions and times to be examined.

In this study, the Semi-solid Control Diagram is a new proposed tool based on previous studies in solid dosage forms. It is used as an instrument for studying and evaluating semi-solid pharmaceutical dosage forms, by comparing several different lipogels and evaluating a number of previously selected parameters, resulting in a simple and rapid method for characterizing and determining which profile is most suitable for achieving a good stability. This tool can also be used in the development of pharmaceutical dosage forms in hospitals, where no pharmaceutical design is done; however, a high quality of the drug products is required to be administrated to the patients. This test could be used as a quality control test for small batches prepared in hospitals.

As a consequence, this tool could be used to save time on the pharmaceutical development.

## Materials and methods

The active substance under study is an anti-inflammatory API (Active Pharmaceutical Ingredient) suitable for topical use.

The excipients that have been used for the study are Colloidal Silicon Dioxide, (Aerosil^®^ 200, Fagron, Spain), Medium-chain Triglycerides (Fagron, Spain), Butylated Hydroxytoluene (Fagron, Spain) and Polysorbate 80 (Tween^®^ 80, Fagron, Spain).

### Product characterization using the Semi-solid Control Diagram (SSCD)

The Semi-solid Control Diagram (SSCD) is based on the experimental study and quantitative determination of the characterization parameters of semisolid formulations, being the main objective, the determination of the adequacy of the formula respect to its physical quality. The main contribution of the method is that the study of these parameters individually provide a very partial information of the system, however studying them together we can get a prediction most accurate about the quality of the product.

The new design for semisolid products is based on converting the experimental values obtained from the galenic tests performed on the developed formulations into radius values, using validated formulas that yield a normalized diagram.

In order to verify the control and characterization of the semisolid products, the radius of the Semi-solid Control Diagram (SSCD) for each parameter are determined.

The system parameters are as follows:

Organoleptic properties (Corg)
Viscosity (ŋ)
Extensibility (ε)
Water activity (a_w_)
Centrifugation (Cf)

The first three parameters define the physical and technological properties of the semisolid products.

Water activity indicates the free or fixed water content of the semisolid products that is able to react and that may affect the properties or the stability of the pharmaceutical dosage form or the active principle it contains.

Centrifugation tests the robustness of the semisolid products and the stability of the pharmaceutical dosage form, evidenced by the absence of phase separation or fluidization under conditions of mechanical stress.

The parameters will be obtained in accordance with the described methodology and will be converted to diagram radius by applying the equations proposed.

In summary, by using these experimental physical tests we are able to establish validated measures that determine the quality of the formulated product and allow us to compare the different semisolid products references in order to determine which formulation will present the best results, keeping in mind its intended use for topical applications.

### Parameters of Semi-solid Control Diagram (SSCD)

The tool SSCD consists of 5 parameters, each parameter can be divided into others. These components are described in the following sections.

#### Organoleptic properties (Corg.)

In this case it has been divided into 5 attributes measured by 5 qualitative tests, and it has been considered that all have the same weight in the final radius of the parameter (1/5), because none was considered more prevalent over the rest (based on practical experience in our laboratory).

In the absence of a quantifiable value of each qualitative test, the assignment of a value between 0 and 2 has been directly established in order to contribute to obtaining the radius final value of the organoleptic characteristics properties, and to facilitate the quantification of this radius.

Visual inspection of the sample and overall appearance is performed by placing a sample amount on a glass plate. The radius final value for organoleptic properties parameter is determined by adding together the experimental values (eV) of each of the five following properties: homogeneity, color, flow, absence of air and texture.

- *Homogeneity* (for the sample on the glass plate): limit value (eV) = 2
  The criteria for maximum homogeneity (2) is no physical discontinuities (oil, water) or no clumps visible in the sample with the naked eye and under a microscope.
  If small discontinuities appear (only visible under a microscope, not with the naked eye), a value of 1 is assigned.
  If some discontinuities are visible with the naked eye, a value of 0 is assigned.
- *Color*: limit value (eV) = 2
  The color is checked with the naked eye.
  If color is uniform throughout the sample: 2.
  If there are non-uniform parts, but they are almost imperceptible: 1.
  If different shades of color are visible: 0.
- *Flow through a tube or cannula*: limit value (eV) = 2
  The sample’s flow through a cannula with a diameter of 4.80 mm using manual force is studied and its dispersion is observed.
  If it passes smoothly: 2.
  If it flows with some difficulty and force is required: 1.
  If it does not flow or excessive force is required: 0.
- *Absence of air*: limit value (eV) = 2
  The absence of air is checked with the naked eye.
  If there are absence of air with the naked eye and under a microscope: 2.
  If there are presence of air (only visible under a microscope, not with the naked eye): 1.
  If there are presence of air with the naked eye: 0.
- *Texture (on glass)*: limit value (eV) = 2
  If the texture is as expected and it can be spread properly: 2.
  If the texture is not as expected and it is difficult to spread: 0.

The value of the radius is calculated by adding together the organoleptic valuations with the following formula:

$$eV=r=(P_{1}+P_{2\;}+P_{3\;}+P_{4}+P_{5})$$ (1)

So the maximum value of the radio will be 10 and the minimum value 0.

#### Determination of viscosity (ŋ)

A Brookfield CAP2000 (AMETEK, USA) cone and plate viscometer is used.

A sample amount equivalent to a medium-size droplet is placed in the centre of the viscometer.

The parameters used for the determination are:

- Spindle: 0,4 (24 mm of diameter)
- Temperature: 25 °C
- Hold time: 12 s
- Speed: 400 rpm
- Run time: 12 s

The value of the radius is obtained by applying the following formulas for which, based on experience and on the type of semisolid dosage form established as target, range limits included in the Table 1, can be set to calculate the radius of the developped formulation.

**Table 1:** Types of gel in function of the viscosity.

Type of gel	Limits* (reference viscosity v_1_)	Formula
Low viscosity gel	100–1000 mPa·s	r=10-(v1100-v2100)^a^; ν_1_ = 1000
Medium viscosity gel	1000–10000 mPa·s	r=10-(v11000-v21000)^a^; ν_1_ = 10000
High viscosity gel	10000–100000 mPa·s	r=10-(v110000-v210000)^a^; ν_1_ = 100000

^a^ ν1 = reference viscosity (in mPa·s); ν2 = problem viscosity obtained (in mPa·s)

*Note: The limits established for the viscosity will be determined by the type of semisolid dosage form studied or desired. A medium viscosity gel is the target set for the lipogel and in this case the formula of the second line should be applied to calculate the radius. In this case the target about viscosity (v_1_) will be 10000 mPa·s. If the objective is to obtain a paste semisolid form with high viscosity, in this case the formula of the third line should be applied, and the target v_1_ should be 100000 mPa·s.

#### Determination of extensibility (ε)

**Fig 1:**
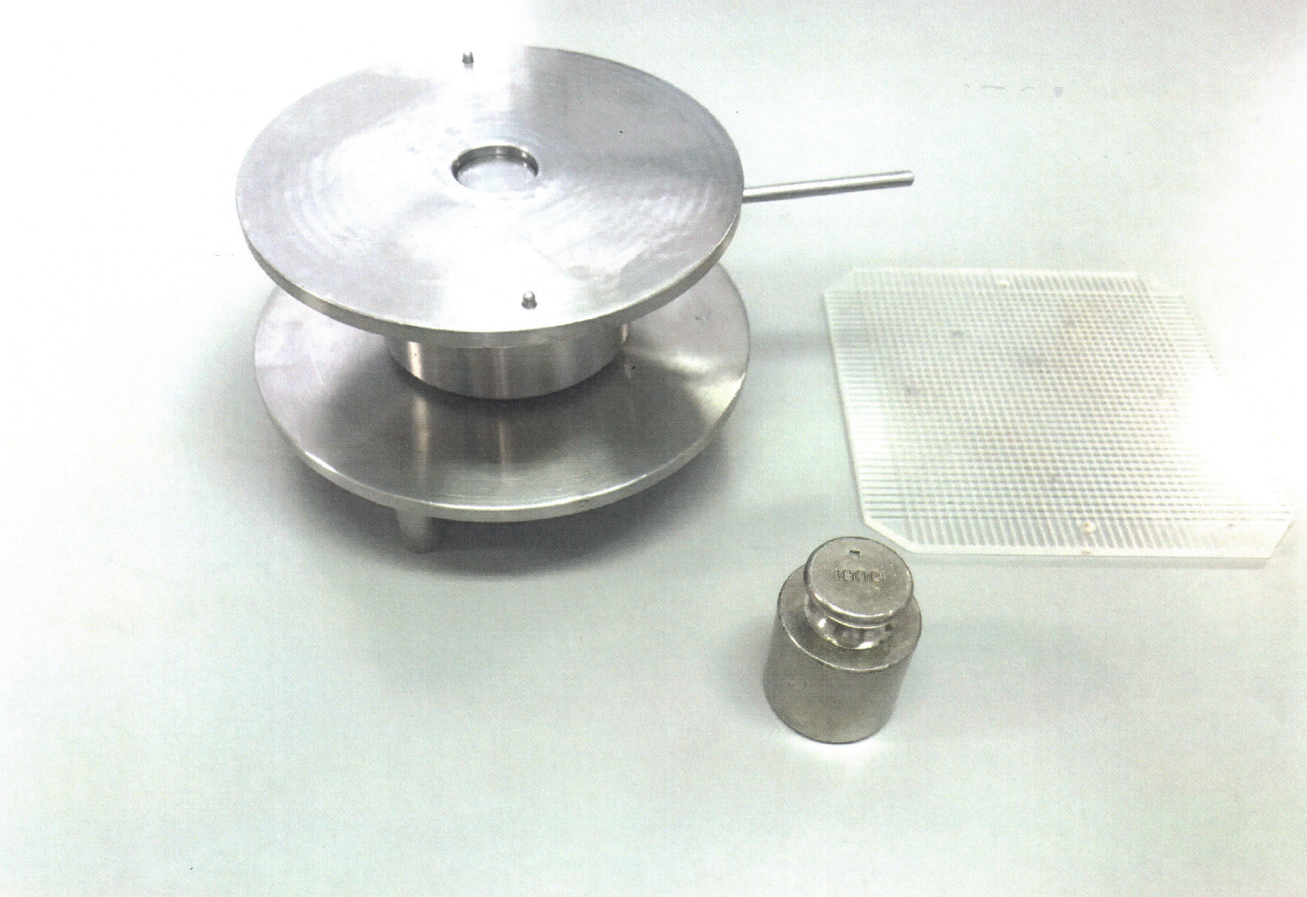
Suñé Arbussà / del Pozo Ojeda strain gauge used to calculate the extensibility.

The SUÑÉ ARBUSSÀ / DEL POZO OJEDA strain gauge is used (Fig 1) [17] [18].

The sample is placed on the strain gauge orifice with a higher of 3 mm, making sure it is full and level. Next, it is covered with a lid and a 100 g weight is placed on it for 1 minute.

As a result of the pressure, the samples extend under the lid in a substantially circular way, so that by measuring two of its perpendicular diameters the average value of the diameter of the theoretical circle can be known and, from this, the surface occupied by the sample under the action of the 100 g weight.

**Fig 2:**
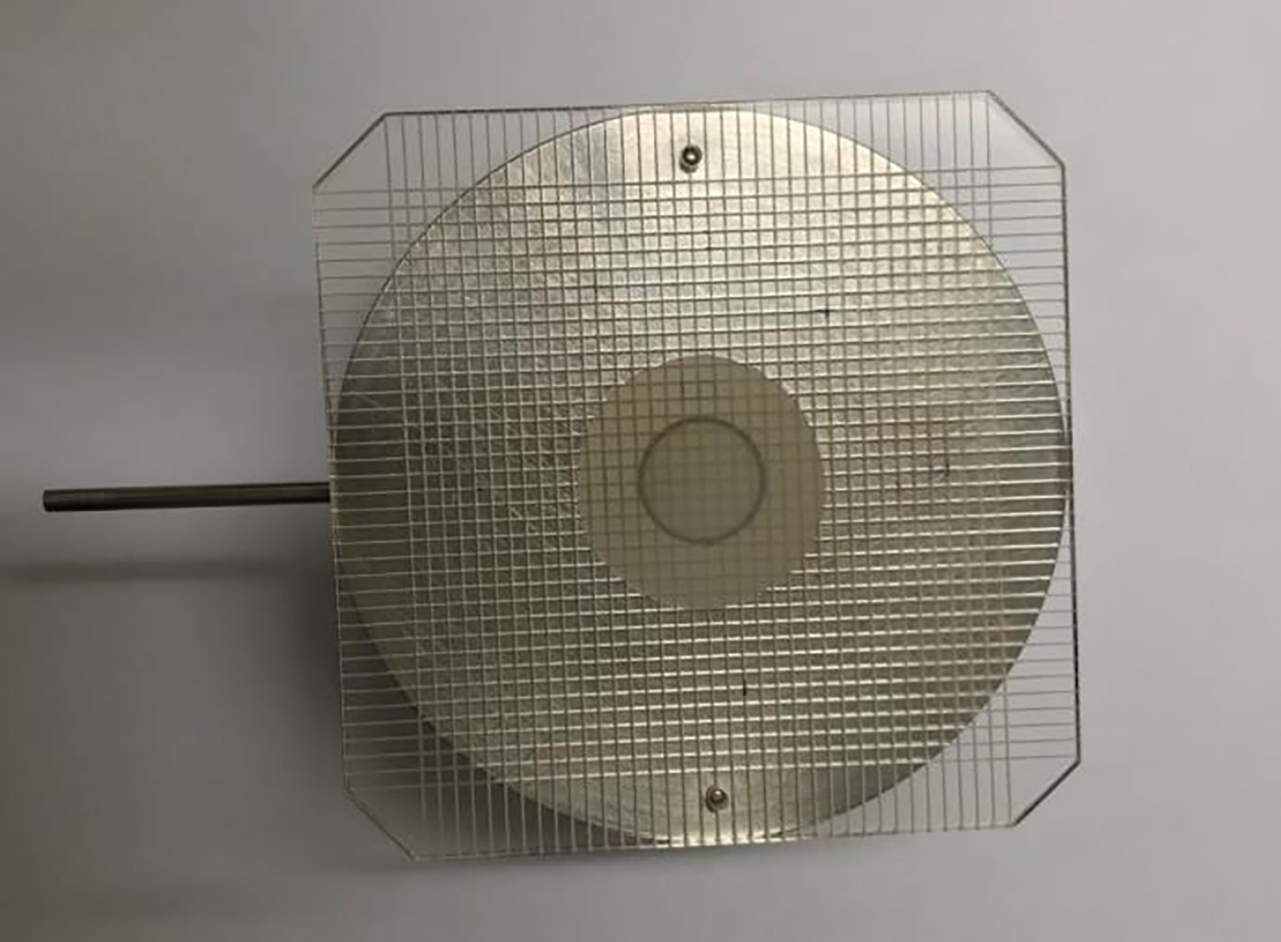
Suñé Arbussà / del Pozo Ojeda strain gauge with the sample.

**Fig 3:**
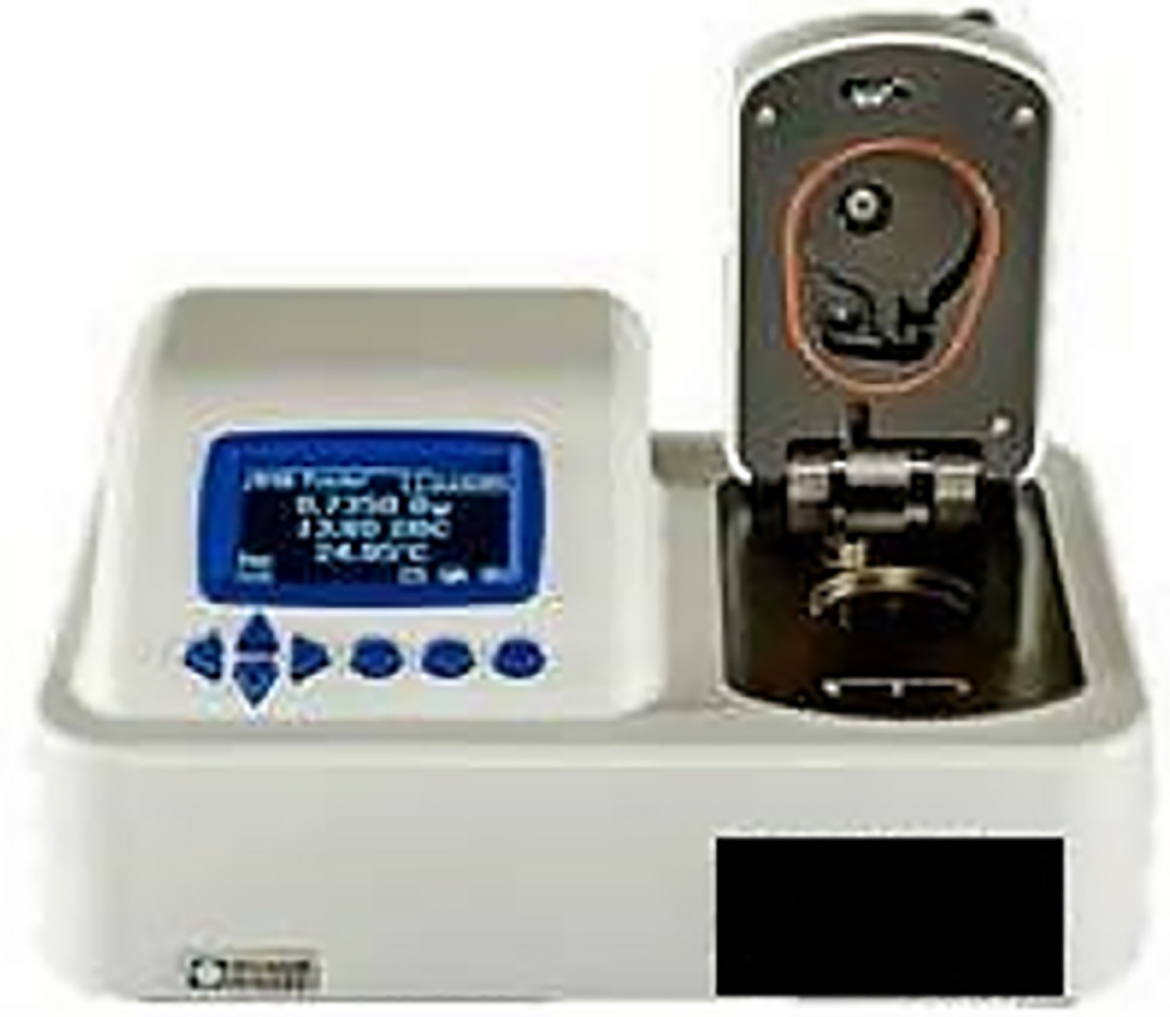
AQUALAB 4TEV equipment.

The diameter obtained is measured using a caliper (Fig 2) and 3 measurements are made and the mean value is calculated. Using this value as the basis, the area of the circle is calculated (S = π·r^2^).

The value of the radius is calculated by applying the following formula:

r=10-(ε1100-ε2100) (2)

with ε1 being between 100 (poor extensibility, target required for pastes or ointment semisolid dosage forms) and 1000 mm^2^ (high extensibility, required for fluid semisolid dosage form) and where: ε1 = reference viscosity; ε2 = problem viscosity. In the studied case, it is required a high extensibility as a target of quality, so, If ε2 >1000 mm^2^, the value of the radius = 10 and if ε2 <100 mm^2^, the value of the radius = 0.

#### Determination of water activity (a_w_)

Water activity describes the thermodynamic energy status of the water in a system. Though not scientifically correct, it may help to picture water activity as the amount of “available” water in a system. It is not a measure of how much water is present in a product, but is an indicator of how much the water in the product resembles and behaves like pure water. Water activity values range from 0 (bone dry) to 1.0 (pure water). As water activity decreases, the water in a product decreases in energy and is less “available” for microbial growth, for chemical reactivity, for moisture migration, and to act as a solvent.

*The water activity (a*_w_) corresponds to the ratio of the partial water-vapor pressure in equilibrium with the product analyzed to the water-vapor saturation pressure in equilibrium with pure water at the same temperature [19].

$$a_{w}=\frac{pF\;(T)}{Ps\;(T)}$$ (3)

Where:

*pF(T)*: is the partial water-vapor pressure in equilibrium with the product analyzed at the temperature T (kept constant during measurement);

*PS(T)*: is the water-vapor saturation pressure in equilibrium with pure water at the same temperature T.

Water activity is therefore a dimensionless quantity between 0, which corresponds to a completely anhydrous sample, and 1, which corresponds to pure salt-free water.

The relative humidity at the equilibrium point is 100 times the water activity.

In order to determine a_w_, the dish device (plastic container) is filled halfway with the sample and placed in the lower chamber. The AQUALAB 4TEV (DECAGON DEVICES, USA) (Fig 3) water activity meter is used to measure the water activity.

The value of the radius is calculated using the following formula:

$$r=10-(10\times a_{w})$$ (4)

#### Centrifugation (Cf)

The P SELECTA MEDITRONIC (JP SELECTA S.A, SPAIN) centrifuge is used with a rotor code 7001082 and with a tube diameter of 25 mm.

Approximately 5 g of sample is weighed in a centrifuge tube at a speed of 5000 rpm for 15 minutes. If phase separation occurs, the test is repeated, reducing the speed to 1000 rpm. The radius for this parameter is determined using the values obtained after centrifugation, in Table 2 could be found the assigned values.

**Table 2:** Conversion of results after centrifugation into radius values (*r*).

Results after centrifugation	Radius value
With no phase separation at 5000 rpm/15 min	r = 10
With no phase separation at 1000 rpm/15min	r = 5
With phase separation at 1000 rpm/15 min	r = 0

#### Calculation of the index for the model

As a summary, the list of sub-parameters in order to obtain the value of the five main parameters are shown in Table 3 and the formula to correct the experimental results into the standardized radii is shown in the last column.

**Table 3:** Conversion of limits of each parameter into radius values (r).

Parameter		Limit value	Conversion to radius
**Organoleptic properties**	Homogeneity (P_*1*_)	0–2	*eV* = *r* = (*P*_1_ + *P*_2_ + *P*_3_ + *P*_4_ + *P*_5_)
	Color (P_*2*_)	0–2	
	Flow through a tube or cannula (P_*3*_)	0–2	
	Absence of air (P_*4*_)	0–2	
	Texture (P_*5*_)	0–2	
**Viscosity**		100–100000 mPa·s* (1000 mPa·s)	r=10-(v1100-v2100)
**Extensibility**		100–1000 mm^2^* (1000 mm^2^)	r=10-(ε1100-ε2100)
**Water activity**		1–0	*r* = 10 − (10 × *a*_*w*_)
**Centrifugation**	With no phase separation at 5000 rpm/15 min	10	r = 10
	With no phase separation at 1000 rpm/15min	5	r = 5
	With phase separation at 1000 rpm/ 15 min	0	r = 0

*Note that the limit value of the parameters for viscosity and extensibility to be applied could be different depending on the quality target of the semisolid dosage form. In this case the target required has been specified in brackets.

Finally, the Semi-solid Control Diagram (SSCD) also calculates the following three indexes, which will give easily an idea of the quality of the developed formulas and helps to compare between them.

- ***PI (Parametric Index)***: is calculated using the following formula:
  PI=nofparameterswithavalue≥5/n°oftotalparameters (5)
  Where:
  n° of parameters with a value≥5, indicates the number of parameters whose value is equal to or higher than 5;
  n° of total parameters indicates the total number of parameters studied;
  Limit of acceptance: >0,5 and maximum value: 1
- ***PPI (Parametric Profile Index)***: Average of r for all parameters; the formula described below is applied:
  PPI=∑rn°×r (6)
  The acceptability limit would correspond to: higher than 5 and maximum value of 10.
- ***GQI (Good Quality Index)***: calculated using the following formula:
  GQI=PPI×f (7)
  Limit of acceptance: higher than 5 and maximum value of 10.
  f = Reliability factor = Polygon area/Circle area. In this case is 0.75.

**Fig 4:**
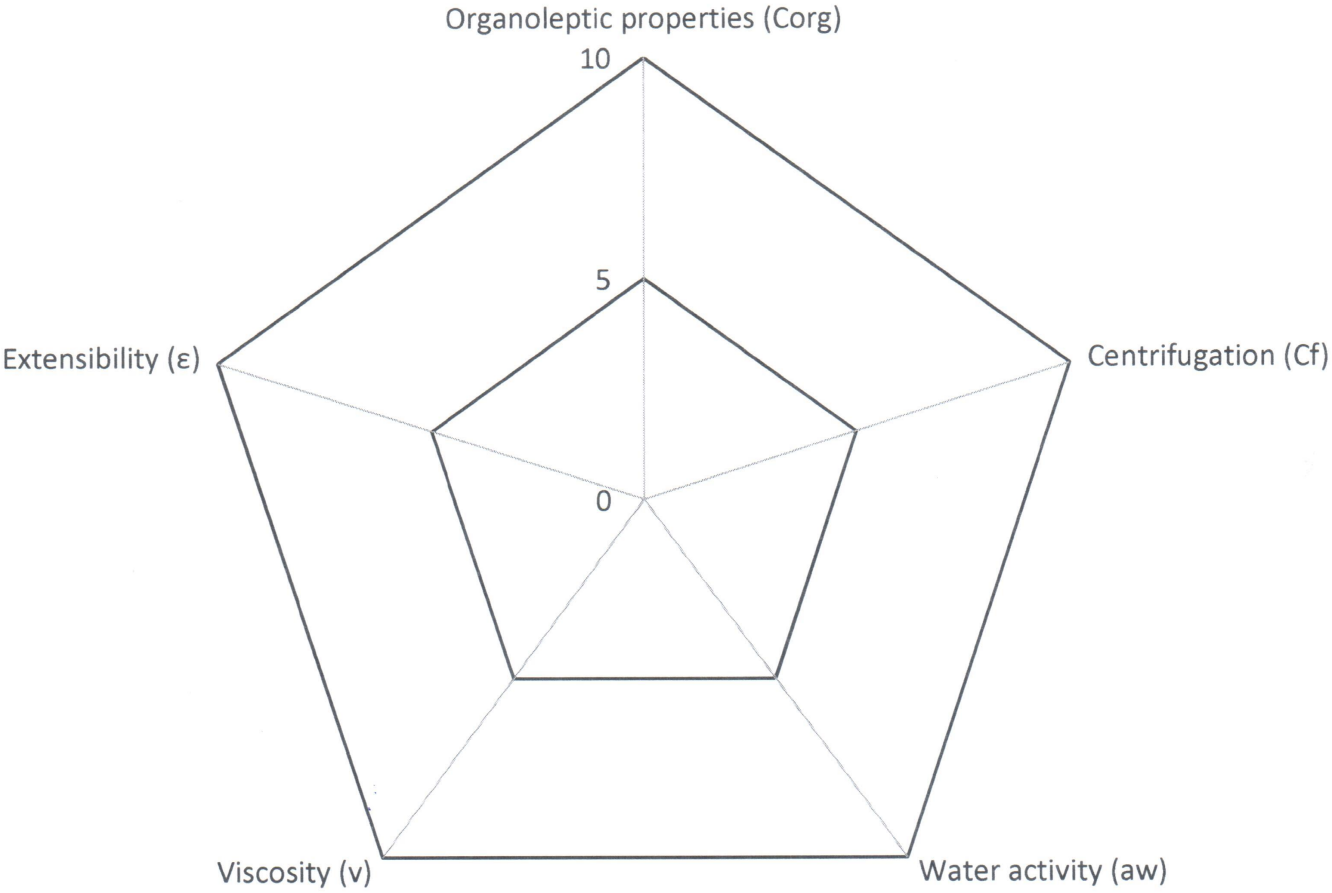
Radius diagram obtained with the application of Semi-solid Control Diagram (SSCD).

The tool likewise provides us a graph of radius where it is possible to visualize easily the properties of the formulation that has been developed (Fig 4).

A quality formula is considered to be one that occupies almost half of the graph’s area or, in other words, one with a GQI of 5. This same tool can be used to evaluate the stability of the formula: the same quality tests are applied after subjecting the formula to a stress process.

### Preparation of the different formulations

Given the characteristics of the active substance to formulate, it is decided to choose an oily base with different coadjuvants.

Three formulations have been developed as shown in Table 4.

**Table 4:** Composition of Reference A1, A2 and A3.

	Reference A1	Reference A2	Reference A3
Composition	%	%	%
API	2.5	2.5	2.5
Colloidal Silicon Dioxide	6.0	6.0	6.0
Butylated Hydroxytoluene	_	0.1	_
Polysorbate 80	_	_	2.0
Medium-chain Triglycerides	Sufficient quantity per 100	Sufficient quantity per 100	Sufficient quantity per 100

Different stability conditions have been applied and each condition has been evaluated by the Semi-solid Control Diagram (SSCD):

- **Finished product controls**: The Semi-solid Control Diagram (SSCD) is applied 24 hours post manufacturing.
- **Stress**: There are no applicable regulations to define a stress conditions in semi-solid formulations. In this case, the Semi-solid Control Diagram (SSCD) is applied after the submission of the lipogel to different temperature changes as (internal laboratory conditions for stress assays) it is explained as follows:
  *First step*: 15 days at 30 °C (The sample is submitted to the Accelerated condition of Climate Zone II)
  *Second step*: 2 days at 5 °C (The sample is submitted to a low temperature).
  *Third step*: 2 days at 22 °C (The sample is submitted to the Long Term Stability condition of Climate Zone II)
  *Fourth step*: Centrifugation at 1000 rpm during 15 minutes (The sample is submitted to a physical stress)
  *Fifth step*: 2 days at 5 °C (The sample is submitted to a low temperature).
  *Sixth step*: 3 days at 30 °C (The sample is submitted to the Accelerated condition of Climate Zone II)
  *Seventh step*: 2 days at 22 °C (The sample is submitted to the Long Term Stability condition of Climate Zone II)

#### Lipogel preparation

The solid active substance is sieved through a 90 μm sieve. The Medium-chain Triglycerides are heated until 80 °C with a heater plate. The Colloidal Silicon Dioxide is added and the two components are mixed with mechanical stirring at 300 rpm. The mechanical stirring is maintained until obtaining a homogeneous lipogel. The rest of components are added and the lipogel is led to cool down.

## Results and discussion

### Semi-solid Control Diagram (SSCD) results for the three formulations

#### Formulation A1

**Fig 5:**
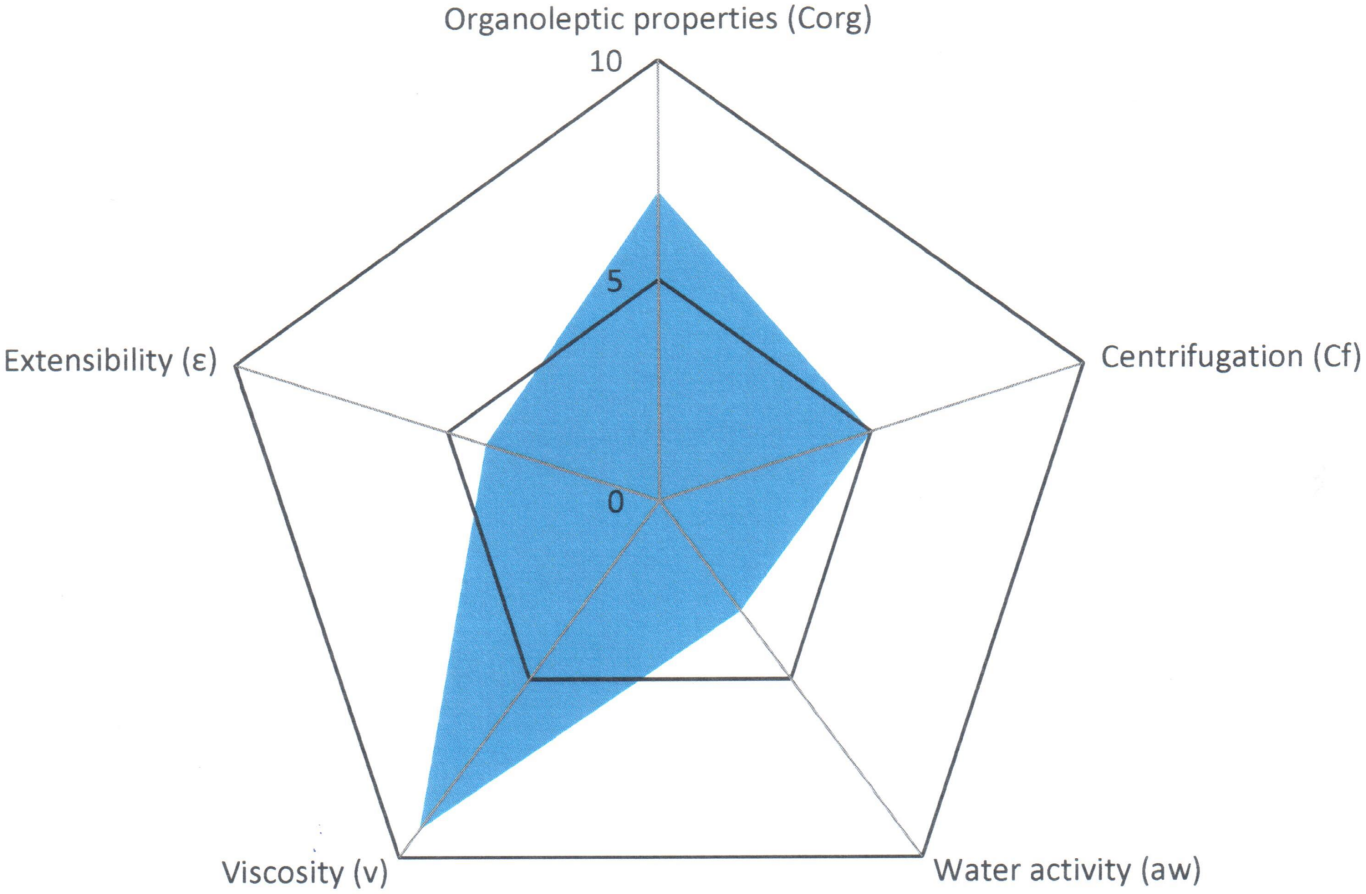
Semi-solid Control Diagram obtained for Reference A1.

Tables 5 and 6 show the Semi-solid Control Diagram results for Reference A1 for the finished product and Fig 5 shows the diagram obtained. (S1 Notebook)

**Table 5:** Semi-solid Control Diagram results for Reference A1 (Finished product and product subjected to stress conditions). The different values obtained from the same assay have been marked in bold.

	Parameters	Limit value	Experimental value	RSD (%)	Conversion to radius	Radius value
**Finished product**	**Organoleptic properties (Corg)**	0–10	7.0	_	eV = *r* = ∑ (*C*.*org*)	7.0
	**Viscosity (v)**	10000 mPa_·_s (from table1)	9234.3	3.20	*r* = 10-[(*ν1*-*ν2)*/1000]	**9.2**
	**Extensibility (ε)**	1000 mm^2^	411.1	7.70	*r* = 10-(*ε1*/100-*ε2*/100)	4.1
	**Water activity (a**_**w**_**)**	1–0	0.7	_	*r* = 10-(10·*aw*)	3.1
	**Centrifugation (Cf)**	0–10	5.0	_	1000 rpm/15min = 5	5.0
**Stress Conditions**	**Organoleptic properties (Corg)**	0–10	7.0	_	eV = *r* = ∑ (*C*.*org*)	7.0
	**Viscosity (v)**	10000 mPa_·_s (from table1)	5361.3	15.01	*r* = 10-[(*ν1*-*ν2)*/1000]	**5.4**
	**Extensibility (ε)**	1000 mm^2^	444.4	5.91	*r* = 10-(*ε1*/100-*ε2*/100)	4.4
	**Water activity (a**_**w**_**)**	1–0	0.5	_	*r* = 10-(10·*aw*))	4.7
	**Centrifugation (Cf)**	0–10	5.0	_	1000 rpm/15min = 5	5.0

**Table 6:** Index for the model.

Index for the model	Finished product	Stress conditions
**PI (Parametric Index)**	0.6	0.6
**PPI (Parametric Profile Index)**	5.7	5.3
**GQI (Good Quality Index)**	5.1	4.8

The Parametric Index (PI = 0.6), the Parametric Profile Index (PPI = 5.7) and the Good Quality Index (GQI = 5.1) show values that posit that the formulation has moderately acceptable behaviour.

**Fig 6:**
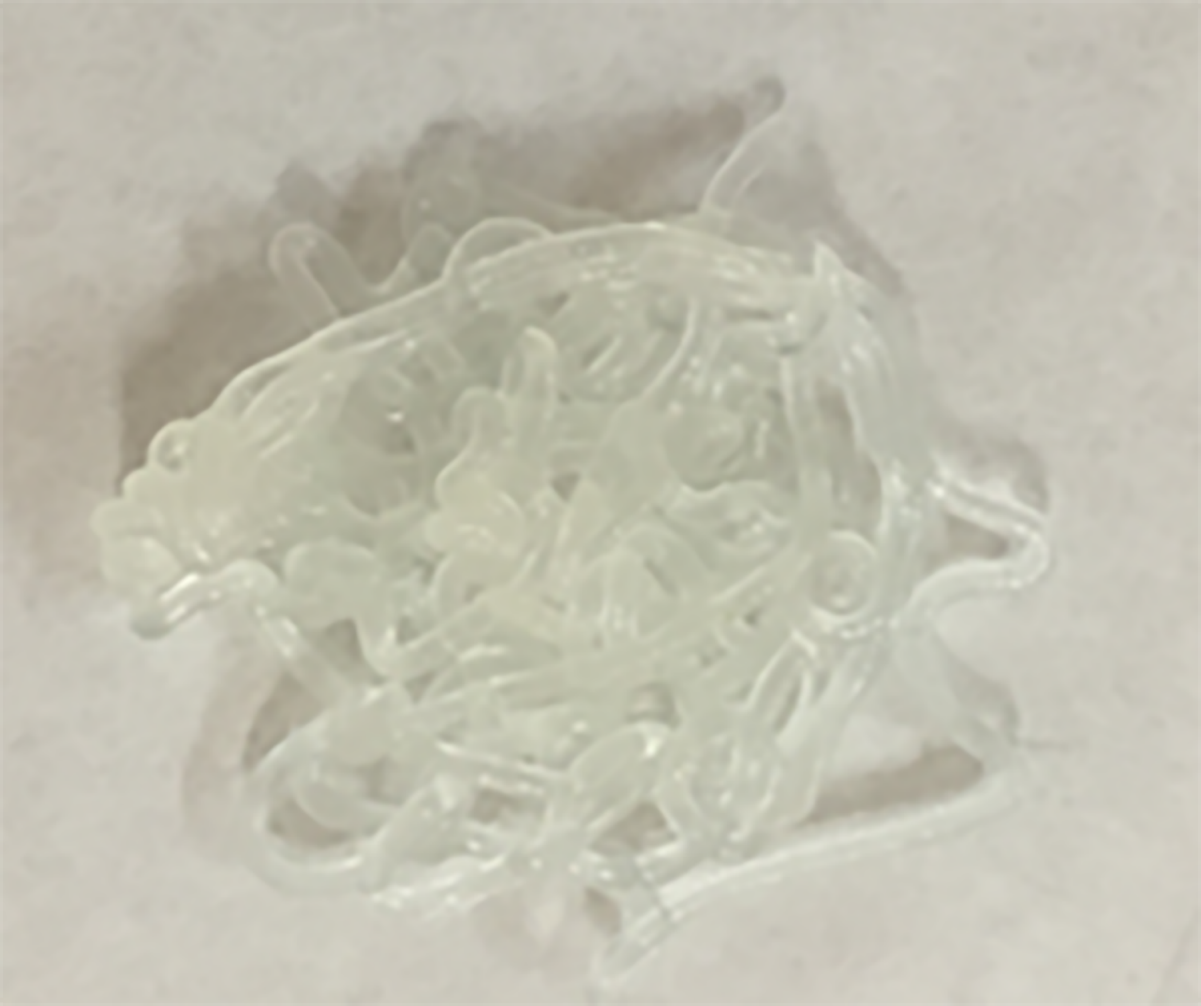
Appearance of Reference A1.

The values of both parameters “Organoleptic properties” (7.0) and “Viscosity” (9.2) are higher than 5, which is more than acceptable with regard to the semi-solid formulation; it meets the specifications required for the project, as far as the appearance (Fig 6). (S1 Appendix)

A value of 5.0 is obtained for “Centrifugation”, which is considered to be correct and sufficient as far as the robustness of the formulation is concerned, but the value for “Water activity” is 3.1, which means that this is a factor that needs to be taken into account in terms of the formulation stability.

The value obtained in “Extensibility” is 4.1, which is slightly lower than the extensibility of the reference standard. Nevertheless, it could be acceptable if all of the other parameters were correct.

**Fig 7:**
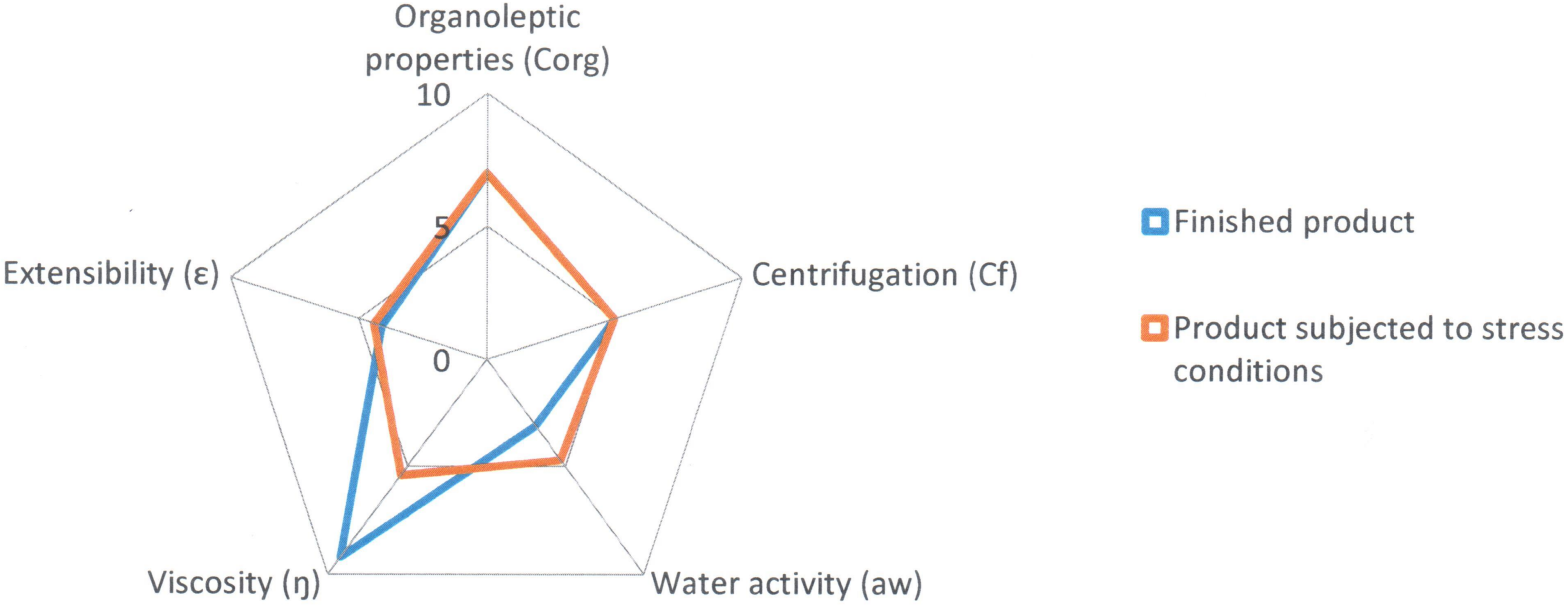
Comparative diagram of Reference A1 (Finished product and product subjected to stress conditions).

After subjecting the lipogel to stress conditions, variations have been observed in the values of some of the radii of the parameters under study. The results of the finished product at time zero and again after being subjected to stress conditions are shown in Table 5 and the comparative diagram of both conditions in Fig 7.

Following the stress test, the Parametric Index (PI = 0.6), the Parametric Profile Index (PPI = 5.3) and the Good Quality Index (GQI = 4.8) show values that are slightly lower than those obtained for the finished product (Table 6).

The “Organoleptic properties” (7.0) and “Centrifugation” (5.0) parameters show the same radius values as before the formulation underwent the stress test.

The “Viscosity” parameter has decreased considerably (5.4), but the values for the “Water activity” (4.7) and “Extensibility” (4.4) parameters have increased somewhat due to the slight loss of water from the formulation caused by the stress to which the sample was subjected.

In Fig 7 we can see that the orange area of the diagram, which corresponds to the stress conditions, has decreased with respect to the finished product (blue area of the diagram), although the difference is not very large (from 5.1 to 4.8).

Thus, it can be concluded that it is foreseeable that the formula will be physically stable, so it could be a good candidate to continue the pharmaceutical development.

#### Formulation A2

**Fig 8:**
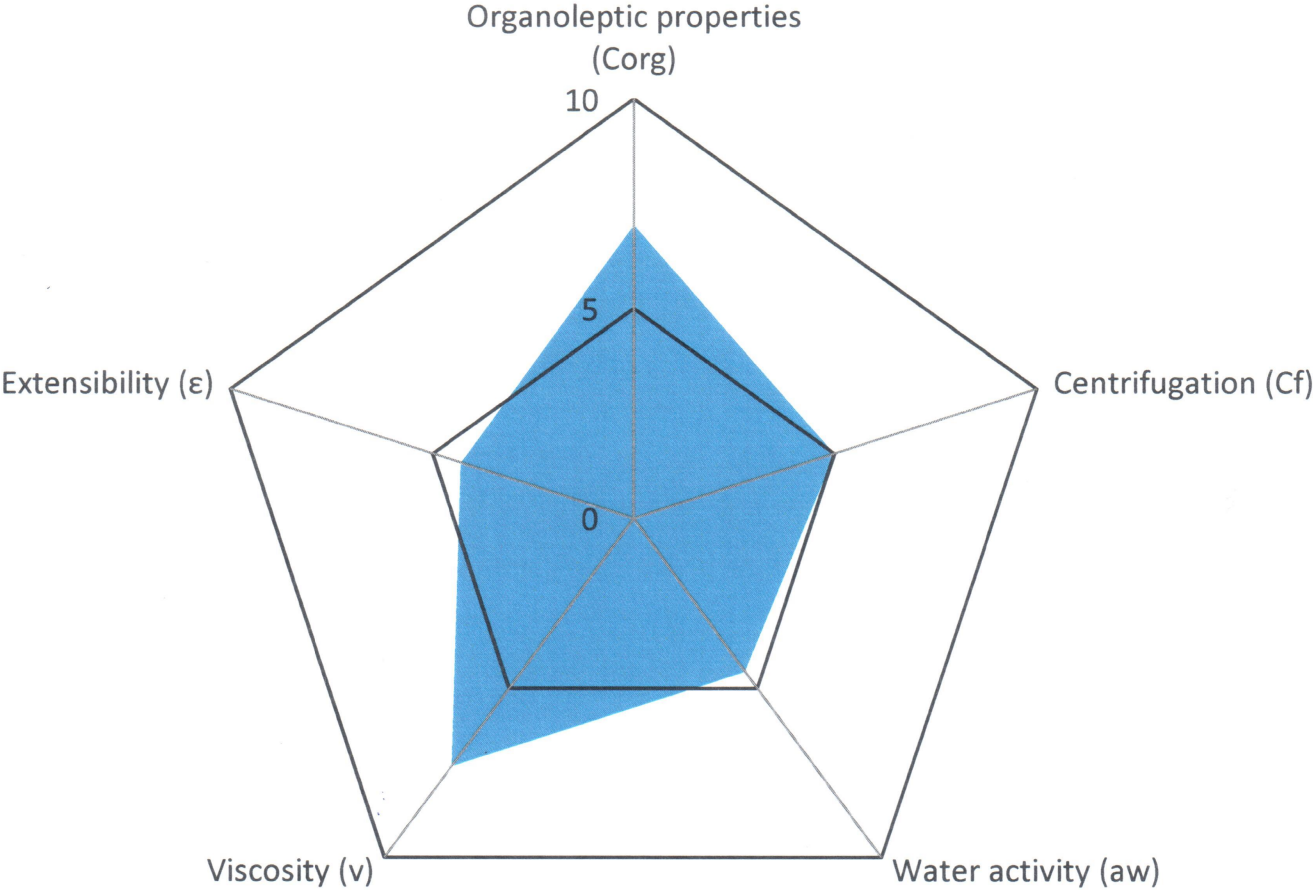
Semi-solid Control Diagram obtained for Reference A2.

Tables 7 and 8 show the Semi-solid Control Diagram results for Reference A2 and Fig 8 shows the diagram obtained. (S2 Notebook)

**Table 7:** Semi-solid Control Diagram results for Reference A2 (Finished product and product subjected to stress conditions). The different values obtained from the same assay have been marked in bold.

	Parameters	Limit value	Experimental value	RSD (%)	Conversion to radius	Radius value
**Finished product**	**Organoleptic properties (Corg)**	0–10	7.0	_	eV = *r* = ∑ (*C*.*org*)	7.0
	**Viscosity (v)**	10000 mPa_·_s (from table1)	7254.0	8.38	*r* = 10-[(*ν1*-*ν2)*/1000]	**7.3**
	**Extensibility (ε)**	1000 mm^2^	433.5	7.78	*r* = 10-(*ε1*/100-*ε2*/100)	4.3
	**Water activity (a**_**w**_**)**	1–0	0.6	_	*r* = 10-(10·*aw*))	4.5
	**Centrifugation (Cf)**	0–10	5.0	_	1000 rpm/15min = 5	5.0
**Stress Conditions**	**Organoleptic properties (Corg)**	0–10	7.0	_	eV = *r* = ∑ (*C*.*org*)	7.0
	**Viscosity (v)**	10000 mPa_·_s (from table1)	4753.0	1.36	*r* = 10-[(*ν1*-*ν2)*/1000]	**4.8**
	**Extensibility (ε)**	1000 mm^2^	501.7	5.44	*r* = 10-(*ε1*/100-*ε2*/100)	5.0
	**Water activity (a**_**w**_**)**	1–0	0.4	_	*r* = 10-(10·*aw*))	5.9
	**Centrifugation (Cf)**	0–10	5.0	_	1000 rpm/15min = 5	5.0

**Table 8:** Index for the model.

Index for the model	Finished product	Stress conditions
**PI (Parametric Index)**	0.6	0.8
**PPI (Parametric Profile Index)**	5.6	5.5
**GQI (Good Quality Index)**	5.1	5.0

The Parametric Index (0.6), the Parametric Profile Index (5.6) and the Good Quality Index (5.1) show values that posit that the formulation will have a moderately acceptable behaviour.

**Fig 9:**
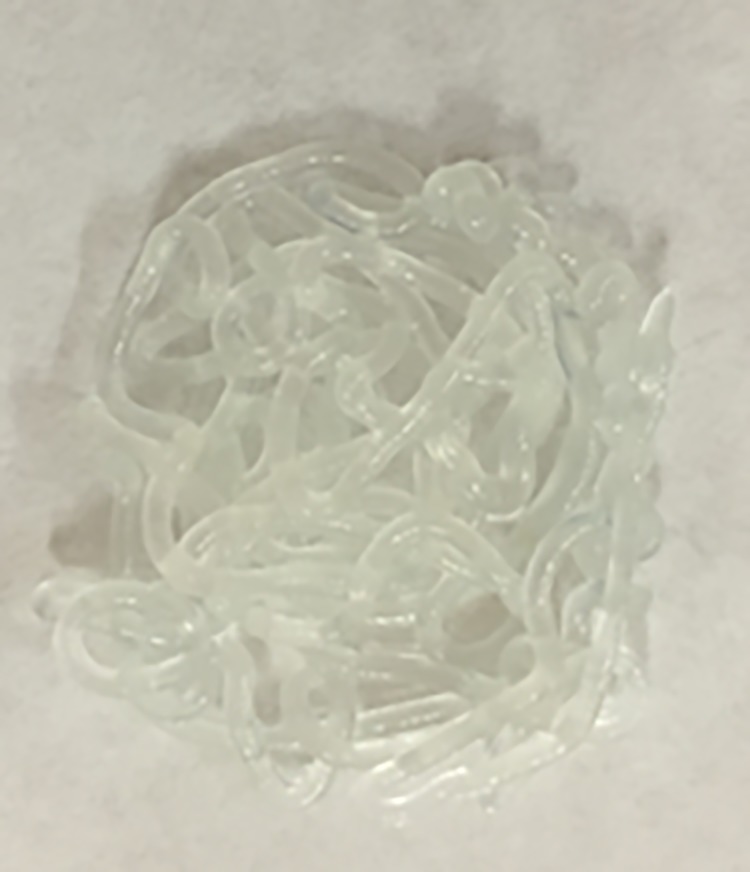
Appearance of Reference A2.

The parameters show values higher than or equal to 5 for “Organoleptic properties” (7.0), “Viscosity” (7.3) and “Centrifugation” (5.0), which are very acceptable as far as the appearance (Fig 9) and robustness of the semi-solid formulation is concerned.

For “Water activity” a value of 4.5 is obtained, which means that this is a factor that needs to be accounted for in terms of the formulation stability. The value obtained for “Extensibility” is 4.3, which is somewhat lower than the extensibility of the reference standard. (S2 Appendix)

**Fig 10:**
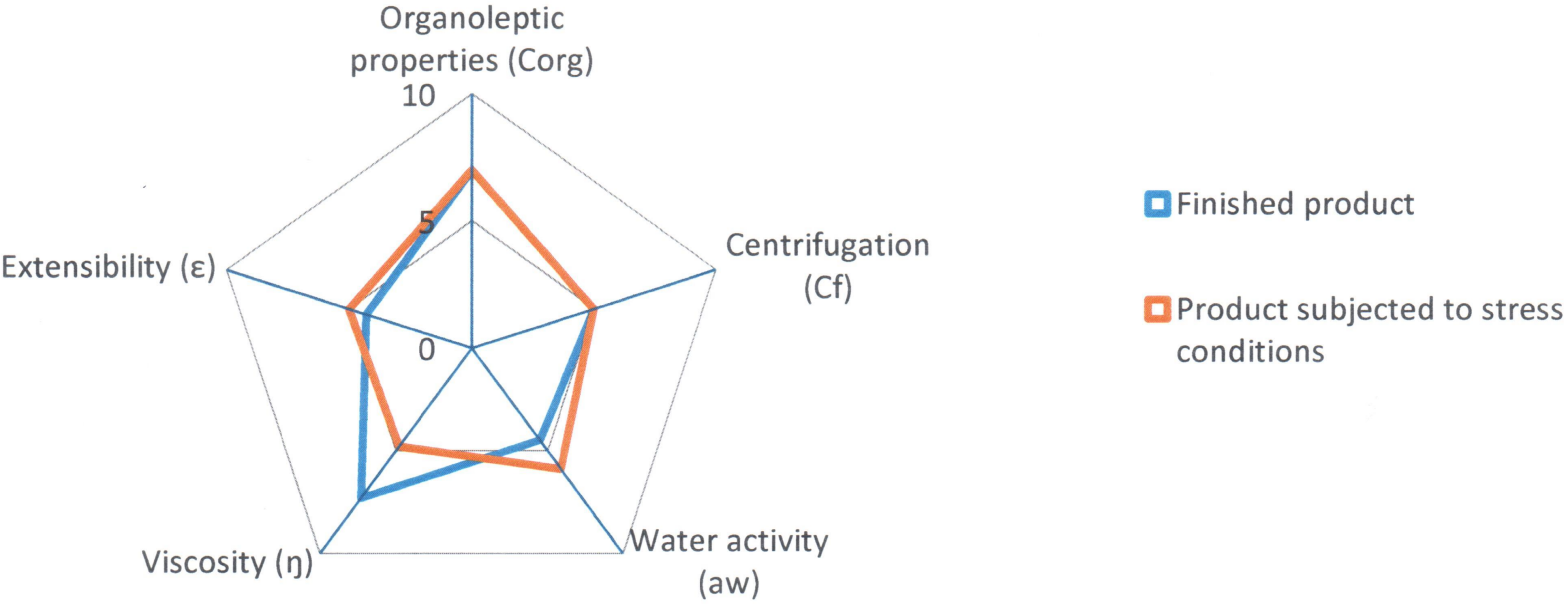
Comparative diagram of Reference A2 (Finished product and product subjected to stress conditions).

After subjecting the lipogel to stress conditions variations have been observed in the values of some of the radius of the parameters under study. The results of the finished product at time zero and again after being subjected to stress conditions are shown in Table 6 and the comparative diagram of both conditions in Fig 10.

The Parametric Index (0.8), the Parametric Profile Index (5.5) and the Good Quality Index (5.0) show values that are similar to those obtained for the finished product (Table 8).

The “Organoleptic properties” (7.0) and “Centrifugation” (5.0) parameters show the same radius values as before the formulation underwent the stress test.

The “Viscosity” parameter has decreased considerably (4.8), but the “Water activity” (5.9) and “Extensibility” (5.0) parameters have increased somewhat due to the slight loss of water from the formulation caused by the stress to which the sample was subjected.

In Fig 10 we can see that orange area of the diagram, which corresponds to the stress conditions, shows an area that is very similar to that of the finished product (blue area of the diagram).

Thus, it can be concluded that it is foreseeable that the formula will be physically stable.

#### Formulation A3

**Fig 11:**
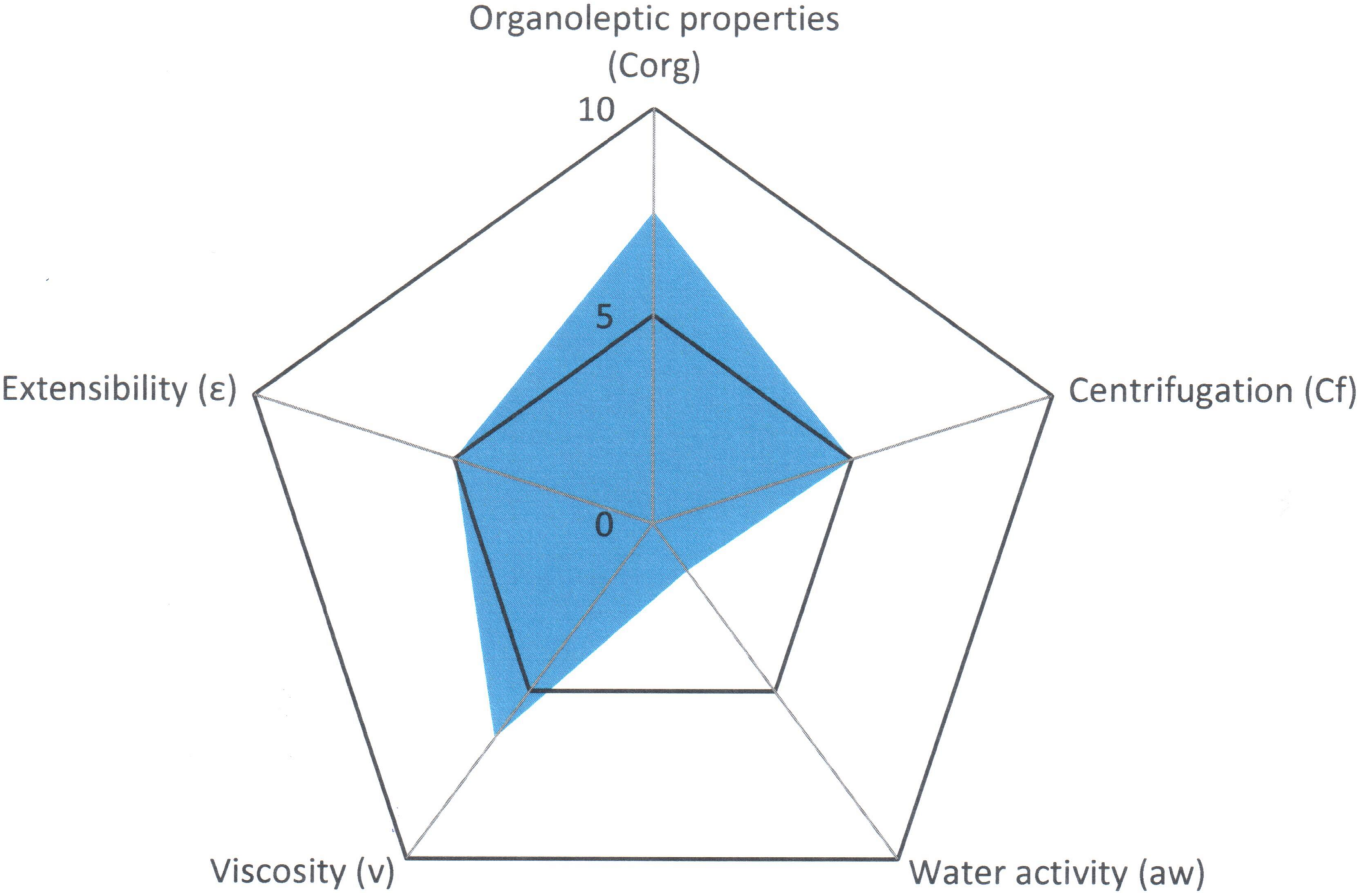
Semi-solid Control Diagram obtained for Reference A3.

Show the Semi-solid Control Diagram results for Reference A3 and Fig 11 shows the diagram obtained. (S3 Notebook).

The Parametric Index (0.8), the Parametric Profile Index (5.0) and the Good Quality Index (4.5) show values that are fairly low, that suggest that the formulation will possibly have an unstable behaviour.

**Fig 12:**
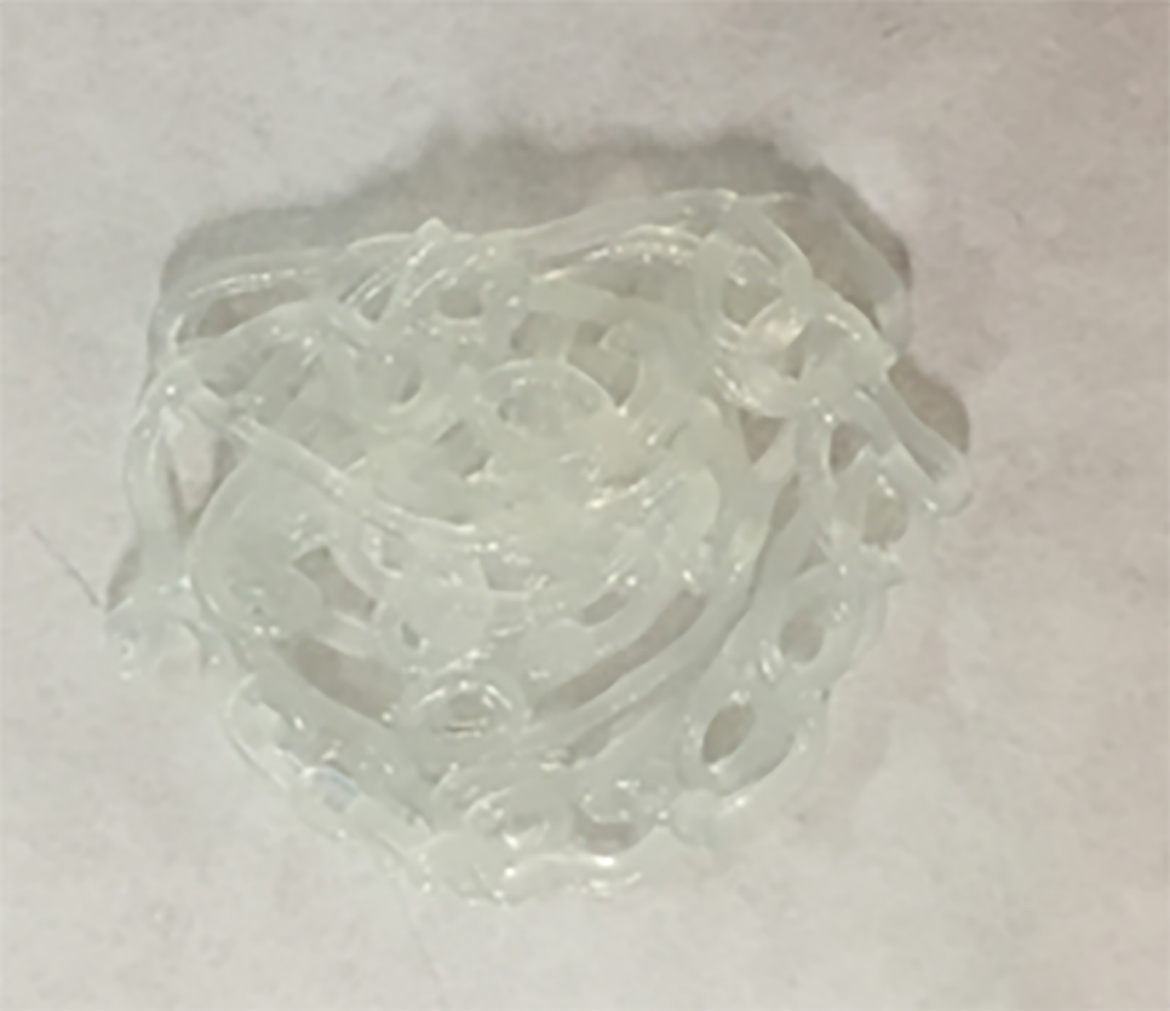
Appearance of Reference A3.

The parameters show values higher than or equal to 5 for “Organoleptic properties” (7.5), “Viscosity” (6.4), “Centrifugation” (5.0) and Extensibility” (5.0), which are correct as far as the appearance (Fig 12) and robustness of the semi-solid formulation.

The value obtained for “Water activity” is 1.4, a very low value, which means that it is a factor that needs to be accounted for in terms of the formulation stability, as the formulation has an elevated free or bound water content with high reactive capacity. (S3 Appendix)

**Fig 13:**
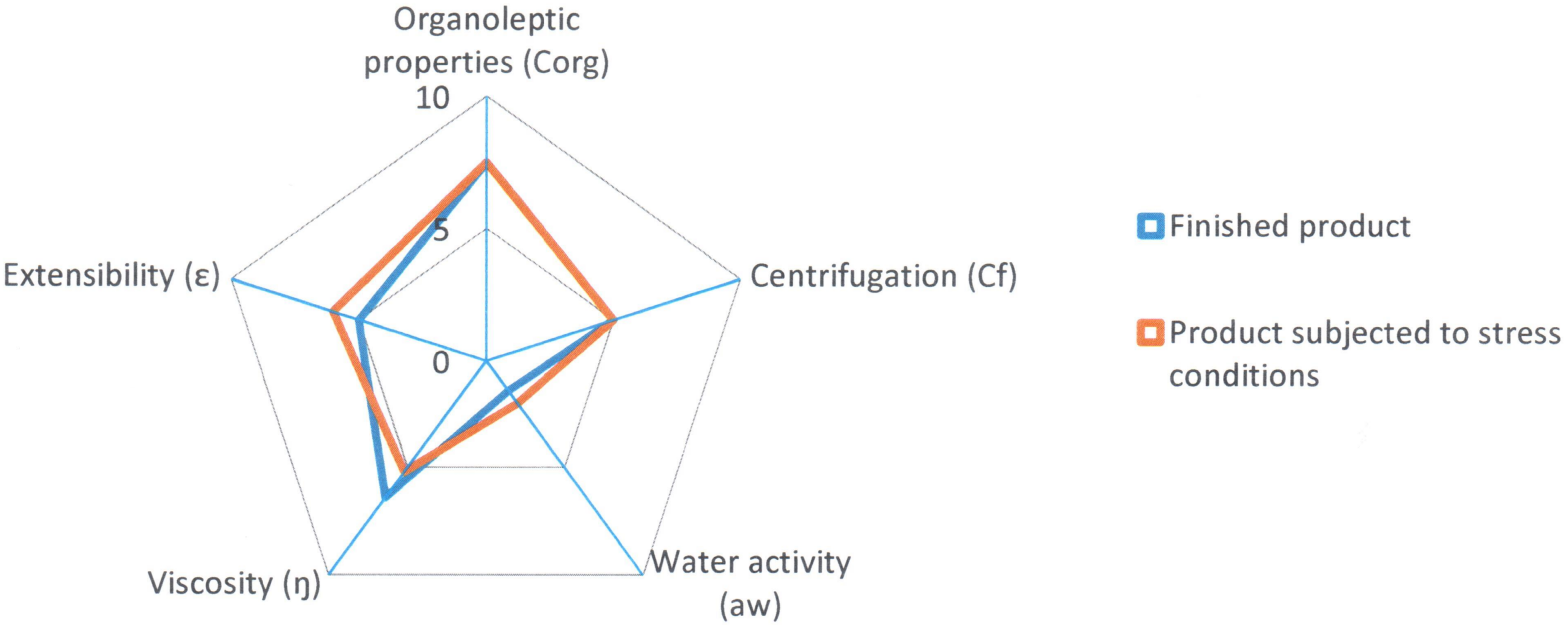
Comparative diagram of Reference A3 (Finished product and product subjected to stress conditions).

After subjecting the lipogel to stress conditions, variations have been observed in the values of some of the radii of the parameters under study. The results of the finished product at time zero and again after being subjected to stress conditions are shown in Tables 9 and 10 and the comparative diagram of both conditions in Fig 13.

**Table 9:** Semi-solid Control Diagram results for Reference A3 (Finished product and Product). The different values obtained from the same assay have been marked in bold.

	Parameters	Limit value	Experimental value	RSD (%)	Conversion to radius	Raddi value
**Finished product**	**Organoleptic properties (Corg)**	0–10	7.5	_	eV = r = ∑ (C.org)	7.5
	**Viscosity (v)**	10000 mPa_·_s (from table1)	6357.8	3.07	*r* = 10-[(*ν1*-*ν2)*/1000]	**6.4**
	**Extensibility (ε)**	1000 mm^2^	500.7	3.88	r = 10-(ε1/100-ε2/100)	5.0
	**Water activity (a**_**w**_**)**	1–0	0.86	_	r = 10-(10·aw))	1.4
	**Centrifugation (Cf)**	0–10	5.0	_	1000 rpm/15min = 5	5.0
**Stress Conditions**	**Organoleptic properties (Corg)**	0–10	7.5	_	eV = r = ∑ (C.org)	7.5
	**Viscosity (v)**	10000 mPa_·_s (from table1)	5214.0	11.90	*r* = 10-[(*ν1*-*ν2)*/1000]	**5.2**
	**Extensibility (ε)**	1000 mm^2^	601.380	4.45	r = 10-(ε1/100-ε2/100)	6.0
	**Water activity (a**_**w**_**)**	1–0	0.79	_	r = 10-(10·aw))	2.0
	**Centrifugation (Cf)**	0–10	5.0	_	1000 rpm/15min = 5	5.0

**Table 10:** Index for the model.

Index for the model	Finished product	Stress conditions
**PI (Parametric Index)**	0.8	0.8
**PPI (Parametric Profile Index)**	5.0	5.2
**GQI (Good Quality Index)**	4.5	4.6

The Parametric Index (0.8), the Parametric Profile Index (5.2) and the Good Quality Index (4.6) show values that are similar to those obtained for the finished product.

The “Organoleptic properties” (7.5) and “Centrifugation” (5.0) parameters show the same radius values as before the formulation underwent the stress test. The “Viscosity” parameter has decreased considerably (5.2) but the “Water activity” (2.0) and “Extensibility” (6.0) parameters have increased somewhat due to the slight loss of water from the formulation caused by the stress to which the sample was subjected.

In Fig 13, it can be seen that the orange area of the diagram, which corresponds to the stress conditions, shows an area that is very similar to that of the finished product (blue area of the diagram).

Thus, it can be concluded that it is foreseeable that the formula will be not physically stable.

### Verification of SSCD tool for a lipogel formulation

Individual comparisons of the results of the Parametric Index, the Parametric Profile Index and the Good Quality Index of References A1, A2 and A3 before and after being subjected to stress conditions show that in every case there are variations in the index, so the Semi-solid Control Diagram tool demonstrates its usefulness in the different stability tests for semi-solid dosage forms.

Similarly, and as described above, this tool can be used to study what effect the addition of certain excipients has on a base formulation and what effects such additions can have on galenic controls.

From a comparison of the three references that were manufactured, it could be concluded that the addition of butylhydroxytoluene in Reference A2 does not modify its Good Quality Index, so it may be considered that adding it will have no impact insofar as galenic controls are concerned.

The addition of Tween^®^ 80 in Reference 3 does indeed involve a more marked decrease of the Good Quality Index, going from an acceptable value to one that is poor (4.5). This could affect the stability of the formulation, so a benefit/risk assessment of adding this excipient should be made.

On the one hand, two of the studied parameters, Viscosity and Extensibility, usually present a wide range of results due to the nature of the assay so it is often difficult to observe significant differences between formulations taking into account these two parameters.

On the other hand, SSCD, by having the ability to evaluate the five parameters together, allows to obtain three different indexes that help us to detect differences between formulations or between excipients that would be difficult or impossible to detect analysing the parameters individually.

These differences help the formulator to discriminate and optimize the process of pharmaceutical development.

To demonstrate improvement in the pharmaceutical development of semisolid pharmaceutical forms and the application of SSCD tool, in Tables 11 and 12 different formulations are compared.

**Table 11:** Semi-solid Control Diagram results for References A1, A2 and A3 (Finished product).

Galenical Parameters usually evaluated	Reference A1	Reference A2	Reference A3
**Organoleptic properties (Corg)**	7.0	7.0	7.5
**Viscosity (mPa·s)**	9234.3	7254.0	6357.8
**Extensibility (mm**^**2**^**)**	411.1	433.5	500.7
**Water activity**	0.7	0.6	0.9
**Centrifugation**	5.0	5.0	5.0
**Index for the model SSCD**			
**PI (Parametric Index)**	0.6	0.6	0.8
**PPI (Parametric Profile Index)**	5.7	5.6	5.0
**GQI (Good Quality Index)**	5.1	5.1	4.5

**Table 12:** Semi-solid Control Diagram results for References A1, A2 and A3 (Product subjected to stress conditions).

Galenical Parameters usually evaluated	Reference A1	Reference A2	Reference A3
**Organoleptic properties (Corg)**	7.0	7.0	7.5
**Viscosity (mPa·s)**	5361.3	4753.0	5214.0
**Extensibility (mm**^**2**^**)**	444.4	501.7	601.4
**Water activity**	0.5	0.4	0.8
**Centrifugation**	5.0	5.0	5.0
**Index for the model**			
**PI (Parametric Index)**	0.6	0.8	0.8
**PPI (Parametric Profile Index)**	5.3	5.5	5.2
**GQI (Good Quality Index)**	4.8	5.0	4.6

For example, fixing us the parameter of viscosity in Table 11, the three values obtained for the three formulations are among 40000 and 5000 mPa*s. In galenical terms, these results are the same but these small differences are reflected in the Good Quality Index.

Therefore, the differences detected between the GQI value of references A1 and A3 (a difference of 0.6) would not have been perceived if the individual parameters had been analysed.

SCDD has allowed to discard the use of one excipient (Tween 80 in Reference A3) since its affects the quality of the formulation. The addition of butylhydroxytoluene in Reference A2 does not affect the quality of the reference so the addition of this would be evaluated by the formulator.

Seeing the stability data of the three references subjected to stress conditions and taking into account the GQI, the Reference A2 can be considered the best formulation. (Table 12)

So, analyzing all the data obtained with SSCD, the development line should be followed from Reference A2 since it has obtained the best indexes of both, finished product and product subjected to stress conditions.

In this way, it is avoided to go ahead with two formulations that do not bring the expected quality to the development.

This tool allows the detection of the quality differences, which indirectly reduces the number of trials to be performed and optimize the costs of the pharmaceutical development.

## Conclusions

From the results obtained from the development of this experimental project, it is concluded that:

1. The Semi-solid Control Diagram (SSCD) could be an excellent tool for the galenic characterization of semi-solid pharmaceutical dosage forms as SeDeM is for solid pharmaceutical dosage forms.
2. The Semi-solid Control Diagram (SSCD) is of great use for formulation studies since it allows the effect of adding different excipients to a base semi-solid formulation to be studied, as well as, it is useful to know the performance of comparative studies of the same excipient from different suppliers.
3. This tool can also be used to perform and track stability studies of semi-solid formulation, as it allows to compare the diagrams made under different conditions and times and their impact on the formulation to be assessed.

Therefore, the SSCD tool is proposed as an adequate tool for the physicochemical control of semisolid pharmaceutical forms. In this article the tool has been described and its suitability has been validated using three similar pharmaceutical forms from an ongoing project.

## Supporting information

S1 Notebook (PDF)

S2 Notebook (PDF)

S3 Notebook (PDF)

S1 Appendix (DOCX)

S2 Appendix (DOCX)

S3 Appendix (DOCX)
