# Supplementary material for: A new design for the review and appraisal of semi-solid dosage forms: Semi-solid Control Diagram (SSCD)
Source: PLoS One. 2018 Sep 7;13(9):e0201643. doi: 10.1371/journal.pone.0201643 (PMC6128454; doi:10.1371/journal.pone.0201643)
Supplement: S1 Notebook — (PDF) [file pone.0201643.s001.pdf]

Ref. A1 PRODUCTO ACABADO

Actividad del agua:

Sala: CG	Temp: 21 ºC	Fecha: 5/12/14	Técnico: FVI

Código aparato: CG50

Realizar 1 determinación a Tº ambiente.

0,6760 → Tª = 25,08 ºC

Centrifugación:

Código aparato: CQ32

Condiciones:

Condición 1: 5000 rpm durante 15 minutos

Condición 2: 1000rpm durante 15 minutos

Resultados:

Condición 1	separación de fases
Condición 2	correcta.

FVS

Proyecto 14/0006

Controles Lipogel

Referencia evaluada: Ref. A1 **Condición evaluada:** Producto Acabado

Características organolépticas:

Homogeneidad (aplicación sobre vidrio)	Aceptable (1)
Coloración	Blanquecina, uniforme (2)
Textura (sobre vidrio)	Correcta (2)
Ausencia de aire	Aceptable (1)
Salida del tubo o cánula	Aceptable (1)

Viscosidad:

Sala: SI	Temp: 22°C	Fecha: 5/12/14	Técnico: FVI

Viscosímetro: Broockfield 2000 CAP Código : CG32

Parámetros:

Spindle: 0,5 Temp: 25°C Hold time: 20s Velocidad: 20 rpm Run time: 12 sg

Hacer 3 determinaciones y determinar la media.

	Valor (mPa*s)	Media
1	9223	9234,3 mPa*s
2	9537	
3	8943	

Extensibilidad:

Sala: SI	Temp: 22°C	Fecha: 5/12/14	Técnico: FVI

Extensómetro Suñé Arbussà/Del Pozo Ojeda Código: CG25

Parámetros:

Hacer 3 determinaciones y determinar la media.

	Diámetro (mm)	Superficie (mm²)	Media
1	23,45	431,89	411,09 mm²
2	21,84	374,62	
3	23,31	426,75	

[illegible]

Controles Lipogel

Referencia evaluada: Ref. A1 **Condición evaluada:** Condiciones estrés

Características organolépticas:

Homogeneidad (aplicación sobre vidrio)	Aceptable (1)
Coloración	Blanquecina, uniforme (2)
Textura (sobre vidrio)	Correcta (2)
Ausencia de aire	Aceptable (1)
Salida del tubo o cánula	Aceptable (1)

Viscosidad:

Sala: ST	Temp: 22°C	Fecha: 04/02/15	Técnico: FVI

Viscosímetro: Broockfield 2000 CAP Código : CG32

Parámetros:

Spindle: 0,5 Temp: 25°C Hold time: 20s Velocidad: 20rpm Run time: 12s

Hacer 3 determinaciones y determinar la media.

	Valor (mPa*s)	Media
1	4455,0	5361,3 mPa*s
2	5993,0	
3	5636,0	

Extensibilidad:

Sala: ST	Temp: 24°C	Fecha: 04/02/15	Técnico: FVI

Extensómetro Suñé Arbussà/Del Pozo Ojeda Código: CG25

Parámetros:

Hacer 3 determinaciones y determinar la media.

	Diámetro (mm)	Superficie (mm^2)	Media
1	24,19	459,58	444,40 mm2
2	24,19	459,58	
3	22,96	414,03	

FVI

Det. A1 CONDICIONES ESTRÉS

Actividad del agua:

Sala: CG	Temp: 24 °C	Fecha: 04/02/15	Técnico: FVI

Código aparato: CG50

Realizar 1 determinación a Tº ambiente.

0,5303 → Tº = 25,03 °C

Centrifugación:

Código aparato: CQ32

Condiciones:

Condición 1: 5000 rpm durante 15 minutos

Condición 2: 1000rpm durante 15 minutos

Resultados:

Condición 1	Separación de fases
Condición 2	Correcta.

[illegible]
