# Supplementary material for: A new design for the review and appraisal of semi-solid dosage forms: Semi-solid Control Diagram (SSCD)
Source: PLoS One. 2018 Sep 7;13(9):e0201643. doi: 10.1371/journal.pone.0201643 (PMC6128454; doi:10.1371/journal.pone.0201643)
Supplement: S2 Notebook — (PDF) [file pone.0201643.s002.pdf]

Controles Lipogel

Referencia evaluada: Ref. A2 **Condición evaluada:** Producto Acabado

Características organolépticas:

Homogeneidad (aplicación sobre vidrio)	Aceptable (1)
Coloración	Amarillento, uniforme (2)
Textura (sobre vidrio)	Correcto (2)
Ausencia de aire	Aceptable (1)
Salida del tubo o cánula	Aceptable (1)

Viscosidad:

Sala: SI	Temp: 22°C	Fecha: 5/12/14	Técnico: FVF

Viscosímetro: Broockfield 2000 CAP Código : CG32

Parámetros:

Spindle: 0,5 Temp: 25°C Hold time: 20s Velocidad: 20 rpm Run time: 12 sg.

Hacer 3 determinaciones y determinar la media.

	Valor (mPa*s)	Media
1	6847	7254 mPa*s
2	6962	
3	7953	

Extensibilidad:

Sala: SI	Temp: 22°C	Fecha: 5/12/14	Técnico: FVF

Extensómetro Suñé Arbussà/Del Pozo Ojeda Código: CG-25

Parámetros:

Hacer 3 determinaciones y determinar la media.

	Diámetro (mm)	Superficie (mm^2)	Media
1	24,11	456,55	433,52 mm^2
2	23,18	422,0	
3	23,18	422,0	

Pct-A2 PRODUCTO ACABADO

Actividad del agua:

Sala: CG	Temp: 21ºC	Fecha: 5/12/14	Técnico: [illegible]

Código aparato: CG50

Realizar 1 determinación a Tº ambiente.

0,5649 → Tº = 25,05ºC

Centrifugación:

Código aparato: CQ32

Condiciones:

Condición 1: 5000 rpm durante 15 minutos

Condición 2: 1000rpm durante 15 minutos

Resultados:

Condición 1	Separación de fases
Condición 2	Correcta.

Controles Lipogel

Referencia evaluada: Ref. A2 **Condición evaluada:** Condiciones estrés

Características organolépticas:

Homogeneidad (aplicación sobre vidrio)	Aceptable (1)
Coloración	Amarillento, uniforme (2)
Textura (sobre vidrio)	Correcta (2)
Ausencia de aire	Aceptable (1)
Salida del tubo o cánula	Aceptable (1)

Viscosidad:

Sala: S1	Temp: 24ºC	Fecha: 04/02/15	Técnico: FVI

Viscosímetro: Broockfield 2000 CAP Código : CG32

Parámetros:

Spindle: 0,5 Temp: 25ºC Hold time: 20s Velocidad: 20rpm Run time: 12s

Hacer 3 determinaciones y determinar la media.

	Valor (mPa*s)	Media
1	4778,0	4753,0 mPa*s
2	4904,0	
3	4577,0	

Extensibilidad:

Sala: S1	Temp: 24ºC	Fecha: 04/02/15	Técnico: FVI

Extensómetro Suñé Arbussà/Del Pozo Ojeda Código: CG25

Parámetros:

Hacer 3 determinaciones y determinar la media.

	Diámetro (mm)	Superficie (mm^2)	Media
1	25,13	495,99	501,65 mm^2
2	26,01	531,34	
3	24,66	477,61	

FVI

REEFAR CONDICIONES ESTRÉS

Actividad del agua:

Sala: CG	Temp: 24ºC	Fecha: 04/02/15	Técnico: FVI

Código aparato: C650

Realizar 1 determinación a Tº ambiente.

0,4003 → Tª = 25ºC

Centrifugación:

Código aparato: 04/02/15

Condiciones:

Condición 1: 5000 rpm durante 15 minutos

Condición 2: 1000rpm durante 15 minutos

Resultados*:*

Condición 1	Separación de fases
Condición 2	Correcta.

FVI
