# Supplementary material for: A new design for the review and appraisal of semi-solid dosage forms: Semi-solid Control Diagram (SSCD)
Source: PLoS One. 2018 Sep 7;13(9):e0201643. doi: 10.1371/journal.pone.0201643 (PMC6128454; doi:10.1371/journal.pone.0201643)
Supplement: S3 Notebook — (PDF) [file pone.0201643.s003.pdf]

Proyecto 14/0006

Controles Lipogel

Referencia evaluada: Ref. AB **Condición evaluada:** Producto Acabado

Características organolépticas:

Homogeneidad (aplicación sobre vidrio)	Bastante homogenea (1,5)
Coloración	Blanquecina uniforme (2)
Textura (sobre vidrio)	Correcta (2)
Ausencia de aire	Aceptable (1)
Salida del tubo o cánula	Aceptable (1)

Viscosidad:

Sala: SI	Temp: 22ºC	Fecha: 5/12/14	Técnico: FVI

Viscosímetro: Broockfield 2000 CAP Código : CG32

Parámetros:

Spindle: 0,5 Temp: 25ºC Hold time: 20s Velocidad: 20 rpm Run time: 12 seg.

Hacer 3 determinaciones y determinar la media.

	Valor (mPa*s)	Media
1	6583,5	6357,8 mPa*s
2	6237	
3	6253	

Extensibilidad:

Sala: SI	Temp: 22ºC	Fecha: 5/12/14	Técnico: FVI

Extensómetro Suñé Arbussà/Del Pozo Ojeda Código: CG25

Parámetros:

Hacer 3 determinaciones y determinar la media.

	Diámetro (mm)	Superficie (mm^2)	Media
1	25,77	521,56	500,73 mm^2
2	25,17	497,57	
3	24,80	483,05	

FVI

Ref-A3 PRODUCTO ACABADO

Actividad del agua:

Sala: CG	Temp: 21 ºC	Fecha: 5/12/14	Técnico: FVI

Código aparato: CG50

Realizar 1 determinación a Tº ambiente.

0,8630 → Tª = 25,07 ºC

Centrifugación:

Código aparato: CQ32

Condiciones:

Condición 1: 5000 rpm durante 15 minutos

Condición 2: 1000rpm durante 15 minutos

Resultados*:*

Condición 1	Separación de fases
Condición 2	correcta.

Controles Lipogel

Referencia evaluada: Ref. A3 **Condición evaluada:** Condiciones estrés

Características organolépticas:

Homogeneidad (aplicación sobre vidrio)	Bastante homogénea (1,5)
Coloración	Blanquecina uniforme (2)
Textura (sobre vidrio)	Correcta (2)
Ausencia de aire	Aceptable (1)
Salida del tubo o cánula	Aceptable (1)

Viscosidad:

Sala: SI	Temp: 24ºC	Fecha: 04/02/15	Técnico: FVI

Viscosímetro: Broockfield 2000 CAP Código : CG32

Parámetros:

Spindle: 0,5 Temp: 25ºC Hold time: 20 s Velocidad: 20 rpm Run time: 12 s

Hacer 3 determinaciones y determinar la media.

	Valor (mPa*s)	Media
1	4600,0	5214,0 mPa*s
2	5201,0	
3	5841,0	

Extensibilidad:

Sala: SI	Temp: 24ºC	Fecha: 04/02/15	Técnico: FVI

Extensómetro Suñé Arbussà/Del Pozo Ojeda Código: CG25

Parámetros:

Hacer 3 determinaciones y determinar la media.

	Diámetro (mm)	Superficie (mm²)	Media
1	28,32	629,91	601,38 mm²
2	27,10	576,80	
3	27,58	597,42	

FVI

Ret. A3 CONDICIONES EXTREMAS

Actividad del agua:

Sala: CG	Temp: 24ºC	Fecha: 04/02/15	Técnico: FVI

Código aparato: CG50

Realizar 1 determinación a Tº ambiente.

0,7909 → Tº = 25ºC

Centrifugación:

Código aparato: CQ 32

Condiciones:

Condición 1: 5000 rpm durante 15 minutos

Condición 2: 1000rpm durante 15 minutos

Resultados:

Condición 1	Separación de fases
Condición 2	Correcta.

FVI
