# Supplementary material for: A new design for the review and appraisal of semi-solid dosage forms: Semi-solid Control Diagram (SSCD)
Source: PLoS One. 2018 Sep 7;13(9):e0201643. doi: 10.1371/journal.pone.0201643 (PMC6128454; doi:10.1371/journal.pone.0201643)

SDM

CARACTERIZACIÓN LIPOGEL. DIAGRAMA VALORES EXPERIMENTALES DETERMINADOS EN EL SDM

Datos primários: Parámetros

Ref.A1	Condiciones:	Finished product	Ref.A1	Condiciones:	Stress conditions
Caracteristicas organolépticas			Caracteristicas organolépticas		
Parámétros	Valor (1-2)	Radio	Parámétros	Valor (1-2)	Radio
Color	2	7	Color	2	7
Textura	2		Textura	2	
Salida tubo	1		Salida tubo	1	
Homogeneïdad	1		Homogeneïdad	1	
Presència aire	1		Presència aire	1	
Centrifugación			Centrifugación		
Rpm màxima<br>Apsecto del semisólido	Valor	Radio	Rpm màxima<br>Apsecto del semisólido	Valor	Radio
	1000	5		5000	5
	5			1	
Potencial de protones			Potencial de protones		
Actividad del agua	Valor	Radio	Actividad del agua	Valor	Radio
	0,7	3,1		0,5	4,7
Reologia			Reologia		
Viscosidad	Valor (mPa*S)	Radio	Viscosidad	Valor (mPa*S)	Radio
	9234,3	9,2		5361,3	5,4
Extensibilitat	Valor (mm2)	Radio	Extensibilitat	Valor (mm2)	Radio
	411,1	4,1		444,4	4,4
**PI**	**0,6**		**PI**	**0,6**	
**PPI**	**5,7**		**PPI**	**5,3**	
**GQI**	**5,1**		**GQI**	**4,8**	

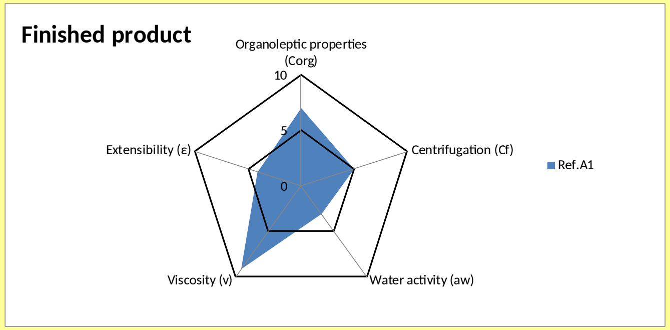

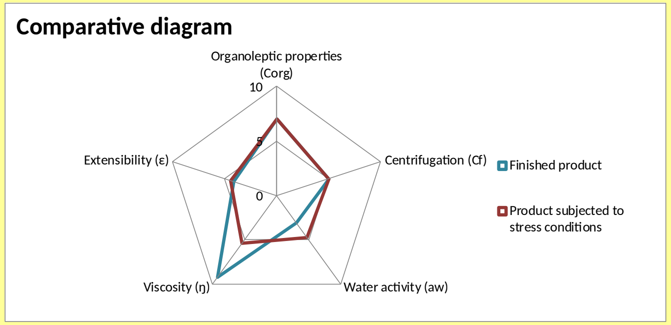

Supplement: Supplementary file 4 [file pone.0201643.s004.docx]
