# Supplementary material for: A new design for the review and appraisal of semi-solid dosage forms: Semi-solid Control Diagram (SSCD)
Source: PLoS One. 2018 Sep 7;13(9):e0201643. doi: 10.1371/journal.pone.0201643 (PMC6128454; doi:10.1371/journal.pone.0201643)

SDM

CARACTERIZACIÓN LIPOGEL. DIAGRAMA VALORES EXPERIMENTALES DETERMINADOS EN EL SDM

Datos primários: Parámetros

**Ref.A2**	Condiciones:	**Finished product**	**Ref.A2**	Condiciones:	**Stress conditions**
Caracteristicas organolépticas			Caracteristicas organolépticas		
Parámetros	Valor (1-2)	Radio	Parámetros	Valor (1-2)	Radio
Color	2	7	Color	2	7
Textura	2		Textura	2	
Salida tubo	1		Salida tubo	1	
Homogeneïdad	1		Homogeneïdad	1	
Presència aire	1		Presència aire	1	
Centrifugación			Centrifugación		
	Valor	Radio		Valor	Radio
Rpm màxima	1000	5	Rpm màxima	5000	5
Apsecto del semisólido	5		Apsecto del semisólido	1	
Potencial de protones			Potencial de protones		
Actividad del agua	Valor	Radio	Actividad del agua	Valor	Radio
	0,6	4,5		0,4	5,9
Reologia			Reologia		
Viscosidad	Valor (mPa*S)	Radio	Viscosidad	Valor (mPa*S)	Radio
	7254	7,3		4753	4,8
Extensibilitat	Valor (mm2)	Radio	Extensibilitat	Valor (mm2)	Radio
	433,5	4,3		501	5
**PI**	**0,6**		**PI**	**0,8**	
**PPI**	**5,6**		**PPI**	**5,5**	
**GQI**	**5,1**		**GQI**	**5,0**	

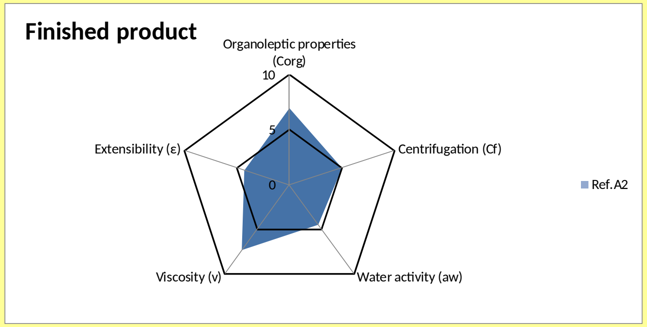

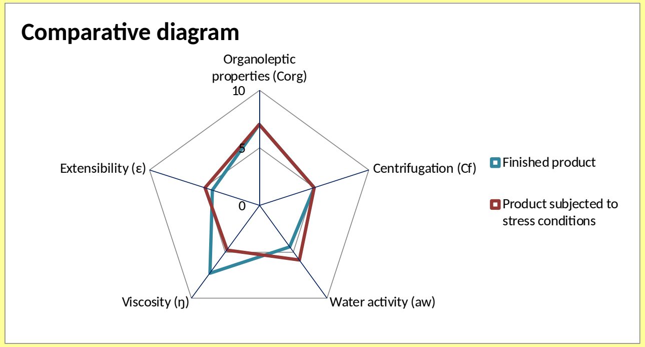

Supplement: Supplementary file 5 [file pone.0201643.s005.docx]
