# Supplementary material for: A new design for the review and appraisal of semi-solid dosage forms: Semi-solid Control Diagram (SSCD)
Source: PLoS One. 2018 Sep 7;13(9):e0201643. doi: 10.1371/journal.pone.0201643 (PMC6128454; doi:10.1371/journal.pone.0201643)

SDM

CARACTERIZACIÓN LIPOGEL. DIAGRAMA VALORES EXPERIMENTALES DETERMINADOS EN EL SDM

Datos primários: Parámetros

Ref.A3	Condiciones:	Finished product
Caracteristicas organolépticas		
Parámetros	Valor (1-2)	Radio
Color	2,5	7,5
Textura	2	
Salida tubo	1	
Homogeneïdad	1	
Presència aire	1	
Centrifugación		
	Valor	Radio
Rpm màxima	1000	5
Apsecto del semisólido	5	
Potencial de protones		
Actividad del agua	Valor	Radio
	0,86	1,4
Reologia		
Viscosidad	Valor (mPa*S)	Radio
	6357,8	6,4
Extensibilitat	Valor (mm2)	Radio
	500,7	5,0
**PI**	**0,8**	
**PPI**	**5,0**	
**GQI**	**4,5**	

Ref.A3	Condiciones:	Stress conditions
Caracteristicas organolépticas		
Parámetros	Valor (1-2)	Radio
Color	2,5	7,5
Textura	2	
Salida tubo	1	
Homogeneïdad	1	
Presència aire	1	
Centrifugación		
	Valor	Radio
Rpm màxima	5000	5
Apsecto del semisólido	1	
Potencial de protones		
Actividad del agua	Valor	Radio
	0,79	2
Reologia		
Viscosidad	Valor (mPa*S)	Radio
	5214	5,2
Extensibilitat	Valor (mm2)	Radio
	601,38	6,0
**PI**	**0,8**	
**PPI**	**5,2**	
**GQI**	**4,6**	

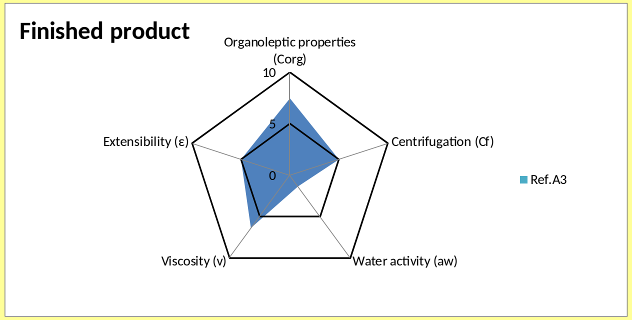

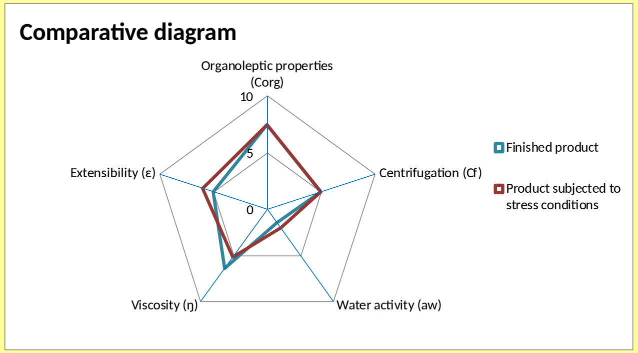

Supplement: Supplementary file 6 [file pone.0201643.s006.docx]
